# Effectiveness of a school-community linked program on physical activity levels and health-related quality of life for adolescent girls

**DOI:** 10.1186/1471-2458-14-649

**Published:** 2014-06-25

**Authors:** Meghan M Casey, Jack T Harvey, Amanda Telford, Rochelle M Eime, Amanda Mooney, Warren R Payne

**Affiliations:** 1 School of Health Sciences, Federation University Australia, Ballarat, Australia; 2 School of Medical Sciences, Discipline of Exercise Sciences, RMIT University, Melbourne, Australia; 3 Institute of Sport, Exercise and Active Living (ISEAL), Victoria University, Melbourne, Australia; 4 School of Education, Faculty of Arts & Education, Deakin University, Melbourne, Australia

**Keywords:** Physical activity, Sport club, Physical education, Adolescent, Female, Effectiveness, Health, Wellbeing

## Abstract

**Background:**

This study evaluated the effectiveness of a school-community program on Health-Related Quality of Life (HRQoL; the primary outcome), physical activity (PA), and potential mediators of PA among adolescent girls living in low-socioeconomic rural/regional settings.

**Method:**

The study was a cluster-randomized controlled trial. Twelve communities with the requisite sports clubs and facilities were paired according to relevant criteria; one of each pair was randomly assigned to the intervention or control condition. Eight schools per condition were randomly selected from these communities and the intervention was conducted over one school year (2011). Female students in grades 7–9 in intervention schools participated in two 6-session PA units – a sport unit (football or tennis) and a recreational unit (leisure centre-based). These were incorporated into physical education (PE) curriculum and linked to PA opportunities for participation outside school. Students were surveyed at baseline and endpoint, self-reporting impact on primary and secondary outcome measures (HRQoL, PA) and PA mediators (e.g. self-efficacy). Linear mixed models for two-group (intervention, control) and three-group (completers, non-completers, control) analyses were conducted with baseline value, age and BMI as covariates, group as a fixed effect and school as random cluster effect.

**Results:**

Participants completing baseline and endpoint measures included: 358 intervention (baseline response rate 33.7%, retention rate 61.3%) and 256 control (14.1% and 84.0%). Adjustment for age and BMI made no substantive difference to outcomes, and there were no cluster effects. For HRQoL, after adjustment for baseline scores, the intervention group showed significantly higher scores on all three PedsQL scores (physical functioning: M ± SE = 83.9 ± 0.7, p = .005; psychosocial: 79.9 ± 0.8, p = .001; total score: 81.3 ± 0.7, p = .001) than the control group (80.9 ± 0.8; 76.1 ± 0.9 and 77.8 ± 0.8). The three-group analysis found intervention non-completers had significantly higher PedsQL scores (84.0 ± 0.8, p = .021; 80.4 ± 0.9, p = .003; 81.7 ± 0.8, p = .002;) than controls (80.9 ± 0.8, 76.1 ± 0.9 and 77.8 ± 0.8). There were no significant differences for any PA measure. Intervention completers had significantly higher scores than non-completers and controls for some mediator variables (e.g. self-efficacy, behavioural control).

**Conclusion:**

Positive outcomes were achieved from a modest school-community linked intervention. The school component contributed to maintaining HRQoL; students who completed the community component derived a range of intra-personal and inter-personal benefits.

**Trial registration:**

ACTRN12614000446662. April 30th 2014.

## Background

Participation in physical activity (PA) is important for physical and mental health [1]. Many adolescents, however, do not participate in sufficient levels of PA and fail to meet age-related PA recommendations to achieve health benefits [2,3]. PA levels generally decline markedly during adolescence, and gender, socioeconomic status (SES), and rurality are consistently associated with PA level. Specifically, girls are less active than boys [4,5], adolescents from socioeconomically disadvantaged communities have lower levels of PA [6], and regional living adolescents often have poorer access to services and facilities which negatively influences PA behaviour [7]. The gender disparity in PA has highlighted the need to develop and evaluate interventions targeting at-risk youth to promote PA participation [8,9]. To date few PA interventions have specifically sought to target adolescents residing in low SES or rural communities [10].

The majority of PA promotion interventions for adolescent girls have been school-based without involvement of the family or community, implemented with a focus on increasing PA via physical education (PE) classes and/or health education strategies, and designed to facilitate participation in PA during school time and/or outside of school [9,11]. In recent years the number of school-based interventions focusing on PA for health has increased considerably, from 23 studies in the 1990s to 94 in the first decade of the 21st century [12]. These interventions have had some success, with short-term improvements in school-based PA, but limited evidence of positive effects on PA outside of school or during leisure time [11,13]. A multicomponent school-based study in Australian secondary schools reported no increase in PA, but reductions in self-reported screen time [14,15]. Consequently, multifaceted interventions that target multiple settings such as schools and communities are considered important for making positive changes to adolescent PA levels [13,16]. However, very few interventions to promote PA among adolescent girls have included a formal school-community link and more research is needed [8,17]. Two such studies which have resulted in positive PA outcomes for adolescent girls have included the Lifestyle Education for Activity Program (LEAP) and Trial of Activity for Adolescent Girls (TAAG) interventions. LEAP increased participation in vigorous-intensity PA; although it was unclear which components of the intervention (i.e. PE, health education, school environment, school health services, faculty/staff health promotion and family/community involvement) facilitated this change [17]. TAAG [8] was a 3-year intervention that linked schools and community agencies (e.g. local health clubs and community recreation centres) to develop and promote PA programs for girls. The TAAG intervention resulted in modest improvements in girls’ PA, but only among girls who had been exposed to the intervention during their entire middle school years (6th to 8th grade) [8]. Both LEAP and TAAG were carried out in the United States and it has been suggested there is a “need for more interventions in different geographical and cultural contexts to have a wider evidence base” (p. 535) [16].

In contrast to the United States, where schools, colleges and universities play a dominant role in the provision of organised sport, in countries such as Australia, United Kingdom, Germany, France, and New Zealand community sporting clubs play a larger role in participation from the recreational to elite level. In Australia specifically, the school (PE, school sport) and the community (e.g. sports clubs, recreation centres) are the most common settings in which organised PA is delivered for adolescents. Further, many community sport and recreation organisations in Australia use the school setting to deliver and promote their sports programs, although few have formal, systematic or evidence-based strategies to link school-based sports programs with local community organisations to facilitate sustained participation opportunities [18]. In other contextual variations, demographic variables such as residential rurality and low SES have not been explored when researching the impact of school and community settings on PA behaviour [9].

This study sought to contribute to real-world evidence to better inform public policy and professional practice aimed at improving health and PA of adolescent girls living in low-SES rural and regional communities. The aim of the study was to evaluate the effectiveness of a school-community linked PA-promotion intervention program targeting adolescent girls living in low-SES Australian rural and regional communities on their Health-Related Quality of Life (HRQoL), levels of PA, and a range of potential mediators of PA (e.g. self-efficacy, perceived sport competence).

## Methods

### Intervention

The intervention involved a school-community linked program conducted over a 12-month period for adolescent girls in grade 7 – 9 living in low-SES Australian rural and regional communities. The intervention was designed in a collaborative manner by members of the research team who drew on the expertise and lived experiences of sports coaches from tennis and football, and community instructors from the YMCA. Ethics approval was obtained from the University of Ballarat Human Research Ethics Committee, the Department of Education and Early Childhood Development (DEECD), and the Diocese of the Catholic Education Office. Details of the program design and implementation instructions have been previously published [19]. The program included a school PE component which incorporated student-centred teaching approaches and behavioural skill development. The PE component involved students participating in two 6-session units each designed as session per week during their ‘normal’ PE class time, which ranged from 57 to 100 minutes. The two units were a sport unit (tennis or football) and a recreational unit (YMCA). The PE classes were delivered in a collaborative manner by PE teachers with the relevant community fitness instructors, and tennis and football coaches, and were linked to a community component that was designed to address previously reported barriers to PA participation. Barriers such as skill level, competence, financial costs and teaching/coaching approaches were identified through ethnographic fieldwork and informed the design of the program [19-21]. The socio-ecological model [22] was the overarching theoretical framework that guided the development of the intervention. This was underpinned by: Social-Cognitive Theory (SCT), which involved incorporating self-management strategies to encourage adolescent girls to be independently active [23] and a capacity-building framework [24] to build the capacity of the teachers and coaches to deliver the program within the schools and community, respectively. Specific capacity building strategies included professional development to introduce the key principles of the planned curriculum and teaching approach. The curriculum and teaching approach drew on the principles of Game Sense [25], an Australian derivative of the Teaching Games for Understanding (TGfU) approach [26], and productive pedagogies [27] in curriculum development, which is further described in Casey et al. [19]. Game Sense was adopted as the pedagogical framework for each of the sports units (tennis and football) and saw the focus placed upon the tactical dimensions of the game, rather than skill performance [28]. Further this pedagogical approach was adopted in the school setting to align with recent developments in community sports club coaching contexts. Productive pedagogies include the dimensions of intellectual quality, connectedness, supportive classroom environment, and working with, and valuing, difference. Particular classroom practices that reflected the key tenets of the productive pedagogies framework were identified and signalled to teachers through the use of particular teaching and learning cues in the lesson plan resources [27].

### Sampling design

Quantitatively, the study was a cluster-randomised trial with participants grouped by schools. There were two conditions (intervention and control) with data collected at baseline and endpoint (one year later). Including a control group was important, as whole-of-community approaches to improving physical activity have often not resulted in absolute improvements, but only in improvements relative to control groups [29].

Government and Catholic secondary schools in rural and regional communities in the Australian state of Victoria that met the following criteria were eligible for inclusion: 1) were below the Victorian median (i.e. 1009) for Socio-Economic Indexes for Areas Index of Relative Socio-economic Advantage and Disadvantage (SEIFA IRSAD) [30]; 2) classified by ARIA + [31] as being inner or outer regional; and 3) had a local recreation facility, tennis club and football club. Following this, communities were matched on population size and Government and Catholic schools within these matched communities were eligible for inclusion. A total of twelve low-SES non-metropolitan communities had the requisite combination of sports clubs and leisure centres for delivery of the community component of the intervention. In one pair of communities, which consisted of the two largest regional cities in Victoria, three schools from each community were (subject to agreement by the schools) randomly chosen for inclusion in the study. For the other five pairs of communities, one school was randomly chosen (subject to agreement) from each community, with the exception that when in one control community no school agreed to participate, two schools were chosen from the closest matched control community. In summary, sixteen randomly chosen schools, eight located in six intervention communities and eight located in five control communities, were recruited into the study. The profiles of intervention and control communities ranged in size from 4,233 to 78,222 persons, were below the Victorian median for SEIFA IRSAD (range: 893 – 993), and were between 115 and 331 km from the state’s capital city.

A cohort sampling design was used [17,32] with individuals’ endpoint measures adjusted for their baseline values, potentially increasing the precision of the analysis [33]. The control condition involved schools going about their usual curricular and co-curricular programming and did not include any engagement strategy beyond those currently employed on a routine basis by the intervention program partners: Tennis Victoria, Football Federation Victoria and YMCA Victoria. Control schools received the intervention resources after end-point data collection.

Power analysis was based on the primary outcome HRQoL measures used in the study - PedsQL 4.0 [34]. For derived PedsQL scales, (explained later within measures) there is evidence that the differences between a healthy sample and a sample identified by parents as having a chronic health condition such as asthma, diabetes or attention deficit hyperactivity disorder or depression correspond to effect sizes in the range 0.52-0.81 [34]; that is medium (0.50) to large (0.80) effect sizes [35]. We conservatively specified a small effect size (0.20) for the difference between intervention and control groups. After adjusting for differences between the groups at the start of the study, assuming moderate correlation over time (r = 0.5), ICC = 0.01 and cluster size of 50 for school-level clustering effects, and an intervention:control allocation ratio of 2:1, with a significance level of 5% and 80% power, then based on an independent samples t test, the required sample size was n = 788 (525 intervention and 263 control).

Based on recruitment and retention rates reported in similar international studies such as Pate et al. [17] and Simon et al. [32], tempered by the researchers’ experience of low recruitment rates in the Australian context due to the ethics requirement to obtain explicit informed written consent from both participants and parents/carers, we assumed recruitment (take-up) rates of 40% (intervention) and 20% (control) and retention rates of 80% in both conditions. This led to a requirement for some 3280 girls (1640 in each condition) to be invited to participate in the study. An average of around 200 girls are enrolled in grade 7 – 9 in rural and regional secondary schools in Victoria; thus 16 secondary schools and their associated communities were recruited to participate in the research.

### Evaluation design

The RE-AIM framework [36] was used to examine the Reach, Effectiveness, Adoption, Implementation and Maintenance (RE-AIM) of the program. The extent to which the program reached the intended targets (Reach), the number of schools and students that adopted the program (Adoption), and the barriers and facilitators to the delivery of the intervention (Implementation) have been reported elsewhere [37]. This study addresses the effectiveness of the program.

Impact and outcome variables were measured at baseline and endpoint using available established reliable and valid self-report measures. The use of self-report to assess health behaviours such as physical activity is considered appropriate for the 12 – 15 year age group [38,39].

### Measures

The self-report survey included several health and behavioural measures outlined below and was a paper-based survey administered by teachers during one school period (typically 45mins). In most cases (unless stated), the measure used was the mean score on a set of related Likert scale items.

HRQoL was measured using the validated tool PedsQL 4.0 Generic Core Scales for Teens aged 13 – 18 [34,39]. Internal consistency reliability (Cronbach’s α) for child self-report scores ranged from 0.80 to 0.88. HRQoL is related to an individual’s health and includes the physical, mental and social health dimensions outlined by the World Health Organization [40]. The 23-item PedsQL includes questions on physical functioning (8 items, e.g. “It is hard for me to walk more than a block”), emotional functioning (5 items, e.g. “I feel sad or blue”), social functioning (5 items, e.g. “Other kids tease me”) and school functioning (5 items, e.g. “It is hard to pay attention in class”) which are used to derive summated scores for physical health, psychosocial health (emotional, social and school functioning) and a total score [41]. The summated scores were transformed to a 0 – 100 scale, with high scores indicating better HRQoL.

The concept of “level of PA” is multi-facetted [2]. We assessed the level of PA in three ways. First, self-reported general PA level was measured using a single 5-point Likert scale item: ‘In general, how physically active would you say you are?’ – ‘not at all’ , ‘a bit’ , ‘moderately’ , ‘very’ , ‘extremely’ [42]. Single-item questions have been shown to be acceptable measures of PA when compared to accelerometry measures [43] and to multiple-item questions or diaries [44]. Second, respondents were also asked to report on how many out of the past seven days they had engaged in moderate (e.g. brisk walking, bike riding, dancing) to vigorous (e.g. activities that make you ‘huff and puff’ like netball, soccer, running, swimming) sport or recreational PA for a total of 60 minutes or more, in accordance with Australian Government’s Physical Activity recommendations for 12 – 18 year olds [45]. We refer to these activities as leisure-time moderate to vigorous physical activity (LTMVPA). Finally, PA was measured using a 24-hr Previous Day Physical Activity Recall (PDPAR-24) questionnaire, which has been validated against 24-h step counts in a sample of Australian adolescents with validity coefficients (correlations) ranging from 0.29 to 0.34, p < .05 [46]. Self-report for PA provides low to moderate validity [47] and is considered appropriate to capture a large participant base at low costs [38]. The PDPAR-24 was modified slightly to reflect the Australian female context (e.g. inclusion of netball). Estimates of the rate of energy expenditure in metabolic equivalents (METs) were derived from the PDPAR data using the Compendium of Physical Activities [48]. MVPA was defined as any activity with a MET ≥ 3.0. The number of 30-minute blocks of LTMVPA and hence the total LTMVPA (min) and total MET-weighted LTMVPA (MET-min) were derived from the responses for leisure-time activities in the PDPAR diary [48].

Perceived sports competence was measured using the items of Harter’s Self-Perception of Athletic Competence scale [49], although the scale was modified to suit the format of the overall survey in this study. Our 5-point scale was similar to the 4-point scale of Wichstrøm [50] which had better reliability, convergent validity and factorial validity than the original scale. Participants were provided with five statements (‘I do very well at all kinds of sports’ , ‘I think I could do well at just about any new PA’ , ‘I feel that I am better than others my age at sports’ , ‘I don’t do well at new sports’ , ‘I do not feel that I am very athletic’) with responses: ‘Disagree a lot’ , ‘Disagree’ , ‘Neither’ , ‘Agree’ and ‘Agree a lot’.

Self-management strategies (SMS) [51], perceived behavioural control (PBC) [52], outcome expectation and outcome expectancy-value [52] and self-efficacy were measured with items previously developed and used with adolescent girls, and which were based on contemporary PA behaviour change theories such as theory of planned behaviour and self-efficacy theory [53]. SMS were measured to determine the extent of behavioural and cognitive strategies adopted by respondents to participate in PA, as behaviour self-management underpinned the program design [19]. The scale included four items relating to cognitive strategies and four items relating to behavioural strategies [51]. A fifth item for behavioural strategies was developed by the research team to examine social support behavioural strategies (‘I have a friend or family member who encourages me to do PA’). Participants responded on a 5-point scale ranging from ‘Never’ (1) to ‘Very often’ (5). PBC was measured using a question from Motl et al. [52]. Participants were asked to respond to one item (‘For me to be physically active during my free time on most days would be…”) on a 5-point scale ranging from ‘Very hard’ (1) to ‘Very easy’ (5); responses to the other three items (e.g. ‘I have all the things I need to be physically active during my free time on most days’) were on a 5-point scale ranging from ‘Disagree a lot’ (1) to ‘Agree a lot’ (5). Outcome expectation and outcome expectancy-value were assessed with questions consisting of: belief statements (e.g. If I were to be physically active during my free time on most days it would help me spend more time with friends) on 5-point scales ranging from ‘Disagree a lot’ (1) to ‘Agree a lot’ (5); and corresponding value statements (e.g. spending time with my friends is…), on 5-point scales ranging from ‘Very unimportant’ (1) to ‘Very important’ (5) [54]. The outcome-expectancy value items were formed as a product of the belief and corresponding value item scores [55].

Self-efficacy was measured using a question developed by Nigg [56] which includes 10 items with responses on a 5-point scale from ‘Not at all confident’ (1) to ‘Extremely confident’ (5) to measure confidence in the ability to persist with PA whenever conditions are not ideal. This measure includes five items specific to adolescents (e.g. ‘How confident are you about participating in sport or PA in the next month when you have homework to do?’) and five more generally applicable items (e.g. ‘When you are tired’).

Enjoyment of a range of activities at school and outside of school was also assessed. Participants were asked to indicate how much they liked participating in competitive sport or PA (e.g. basketball, netball), organised but non-competitive sport or PA (e.g. aerobics, ‘Zumba’ , social tennis), and non-organised PA (e.g. walking, jogging, camping) both at school and outside of school. Enjoyment of a range of sedentary activities outside of school was also assessed including: talking on the phone, chatting, texting, watching TV or DVDs, playing computer games, listening to radio, CD, iPod, surfing the internet, and social networking. Responses to the 12 items were on a 5-point scale from ‘Hate it’ (1) to ‘Love it’ (5). A ‘Don’t know, never tried’ (0) category was also provided. Two scores were derived, for PA enjoyment (six items) and sedentary enjoyment (six items).

Barriers to participation in sport and PA included nine items regarding personal barriers (e.g. ‘I don’t like being physically active because of my body shape’) and seven items relating to organisational/environmental barriers (e.g. ‘Cost of participation’). The list of items were derived from PA studies [57]. Participants were asked how often these barriers kept them from participating in sport and physical activity, and the items were scored on a 5-point scale from ‘Never’ (1) to ‘Very often’ (5).

The influence of family and friends on PA and sport participation was measured using a modified version of questions on family and peer influences which has demonstrated good reliability [57]. In terms of family influence, participants were asked to complete several items assessing social support (e.g. encouragement, role modelling, supervision) by indicating in the past month whether a member of their household had: ‘Encouraged you to do physical activities or play sport’ ; ‘Done a PA or played sport with you’ ; ‘Watched you participate in physical activities or sport’ ; ‘Told you that you are doing well in physical activities or sport’ ; or ‘Been willing to assist you with travel to physical activities or sports after school or on weekends’ . In terms of peer influence, participants were asked with respect to the past month ‘Did you encourage your friends to do physical activities or play sports?’ , ‘Did your friends encourage you to do physical activities or play sport?’, ‘Did your friends do physical activities or play sport with you?’ , ‘Did your friends tell you that you are doing well in physical activities or sports?’. The family and peer influence items were scored on a 5-point scale from ‘Never’ (1) to ‘Very often’ (5).

Baseline body mass index (BMI) was calculated by dividing participant’s self-reported weight (in kilograms) by the square of their self-reported height [kg/m^2^]. Finally, baseline age was calculated as the duration from reported birthdate to reported date of completion of the baseline survey, expressed as decimal years.

### Analysis

‘Intention-to-treat’ principles were adopted in part. The usual intention-to-treat approach to loss to follow up is to make the “worst case” assumption that due to non-compliance the intervention treatment has not been experienced, and to carry forward the baseline value, thereby assuming no change in these cases. However, in the present study the first school-based stage of the intervention was mandatory, and the loss to follow up was due only to failure to comply with reporting requirements. Consequently, the carry-forward approach was considered inappropriate in this study, and so only complete cases were analysed. However, respondents who completed both baseline and follow-up surveys but who reported that they did not complete the second community-based stage of the intervention, were included in the analysis, which is in accordance with intention-to-treat principles.

Quantitative outcome measures and summated scales based on multiple Likert items, together with potential mediators, were initially analysed using linear mixed models (LMM), with the baseline value of the measure as a covariate, group as a fixed effect and school as a random effect in order to allow for the investigation of school-level cluster effects. Models were fitted with and without adjustment for age and BMI. Two sets of analyses were conducted. In the first set of analyses, the group factor was the condition of the study (intervention v control). In the second set of analyses, the intervention group was split into self-reported ‘completers’ (those who had participated in the both the in-school component and out-of-school community component) and ‘non-completers’ (those who had participated in the in-school component only) and a 3-group analysis was undertaken. This analysis was aimed at differentiating between the effects of the two components of the intervention (in-school v out-of-school community components). Paired t-tests were used to further examine the patterns of change from baseline to follow-up within each group. A similar suite of analyses were conducted on two dichotomous outcome measures using logistic regression models fitted by the method of generalised estimating equations, incorporating an examination of school-level cluster effects. No cluster effects were observed therefore results of ordinary logistic regression analyses are reported. McNemar chi-square tests were used to further examine the patterns of change from baseline to follow-up within each group. Analyses were conducted using SPSS version 18.0.

## Results

### Response rate

Eight schools (n = 1755 Year 7 – 9 female student enrolment) accepted the invitation to participate as an intervention school; however one school withdrew part way through the intervention due to unsatisfactory experiences with a community sport and recreation provider and data from this school were excluded from analyses (n = 264 students). A further eight schools accepted the invitation to participate as a control school (n = 2208 enrolment).

Participants who completed both baseline and follow-up surveys included 362 intervention students (recruitment rate at baseline 33.7% of student enrolment, retention rate 61.3% of those recruited) and 259 control students (recruitment rate 14.1%, retention rate 83.4%). For analyses incorporating adjustment for age and BMI, participants who did not provide birthdate or estimates of height and weight were excluded. In accordance with the practice of excluding cases for which data are potentially statistically influential but probably spurious [58,59], those whose estimated BMI lay outside the extreme values for 13 year-old females recorded in accumulated data from direct measurements in 21 studies during the period from 1985–2008, incorporating 70,758 Australian children (Professor Tim Olds, personal communication, 9 July 2009) were also excluded. This resulted in data from 263 intervention and 199 control participants being available for analysis of age and BMI.

For analysis associated with the PDPAR-24, participants whose diaries were incomplete were excluded. Cases with estimated MVPA of more than 256 mins/day (i.e. those who reported more than eight 30-minute blocks), which corresponds to the 99th percentile of responses in the 2007 Australian National Children’s Nutrition and Physical Activity Survey (Prof Tim Olds, personal communication, 14 December 2011), were also excluded. This resulted in data from 136 intervention and 107 control participants being available for analysis of MET-mins and time spent in MVPA, of whom 106 intervention and 80 control participants also provided valid age and BMI data. To control for the possibility of systematic differences in PA levels on different days of the week, the PDPAR-24 measures were also adjusted for day of week, by multiplying each observation by the reciprocal of the ratio of the mean value for the particular day of the week to the grand mean value (the unweighted mean of the means).

### Participant profile

Table 1 presents a summary of participant characteristics at baseline – including demographic data, health measures, PA measures, and indicators and potential mediators of PA – of respondents in intervention (completers and non-completers) and control groups. There was a consistent pattern of somewhat higher levels of all health and PA measures and indicators among completers compared to non-completers and controls. However, only three PA measures showed statistically significant differences. The proportion who reported meeting PA guidelines in the past seven days was higher among the intervention group than the control group (p = .021); and both the PDPAR measures (Mins of LTMVPA and MET-mins of LTMVPA) were significantly higher among program completers than controls (p = .016 and p = .002 respectively), and higher among controls than non-completers (p = .028 and p = .010 respectively). There were also significant differences between groups for three of the potential mediators: enjoyment of sedentary activities, which was significantly higher in the control group than the intervention group (p = .027); and perceived sport competence and outcome expectation, which were significantly higher among program completers than non-completers and controls (p = .001 and p = .039 respectively).

**Table 1:** Participant baseline characteristics

	**Intervention:all n=362**		**Intervention:completers n=91**		**Intervention:non-completers n=271**		**Control n=259**	
**Characteristic**	n	Mean ± SD or percent	n	Mean ± SD or percent	n	Mean ± SD or percent	n	Mean ± SD or percent
**Demographics**								
Age (yr)	350	13.4 ± 0.9	88	13.2 ± 0.9	262	13.4 ± 0.9	238	13.4 ± 0.9
BMI	263	19.9 ± 3.9	66	19.8 ± 4.1	197	20.0 ± 3.8	199	19.6 ± 3.5
Live in two parent households	362	78.7	91	74.7	271	80.1	259	74.5
**Health**								
General health – excellent or good	333	71.2	85	80.0	248	68.1	233	73.0
PedsQL physical functioning score	358	83.9 ± 13.0	90	85.7 ± 12.0	268	83.3 ± 13.2	248	84.5 ± 13.2
PedsQL psychosocial functioning score	357	80.6 ± 14.3	90	82.0 ± 12.9	267	80.1 ± 14.7	247	81.5 ± 13.6
PedsQL total score	357	81.8 ± 13.0	90	83.3 ± 11.7	267	81.2 ± 13.4	248	79.8 ± 15.4
**Physical activity:**								
MET-mins of LTMVPA (24-hr)	136	546.4 ± 489.1	27	955.2 ± 590.2	113	454.4 ± 413.6	107	624.5 ± 515.8
Mins of LTMVPA (24-hr)	136	79.2 ± 66.3	27	123.6 ± 77.6	113	69.2 ± 59.4	107	88.6 ± 67.1
Number of days in past seven with at least 60 min of LTMVPA	358	5.0 ± 1.8	90	5.3 ± 1.7	268	4.9 ± 1.8	259	4.8 ± 1.6
Met PA guidelines in past seven days^1^	358	11.2	90	12.2	268	10.8	259	5.8
Sport club/leisure centre member	358	55.0	90	61.1	268	53.0	256	59.4
**Potential mediators:**								
Perceived sports competence	349	3.37 ± 0.74	87	3.62 ± 0.62	262	3.29 ± 0.75	258	3.37 ± 0.74
Self-management strategies	347	3.42 ± 0.75	86	3.55 ± 0.7	261	3.38 ± 0.76	251	3.48 ± 0.77
Perceived behavioral control	358	3.92 ± 0.66	88	3.94 ± 0.64	270	3.91 ± 0.67	255	3.96 ± 0.59
Outcome expectation	351	36.54 ± 5.53	85	37.84 ± 5.02	266	36.12 ± 5.63	253	36.37 ± 5.37
Outcome expectancy-value	346	152.87 ± 48.21	84	161.73 ± 46.14	262	150.03 ± 48.6	246	152.63 ± 46.73
Self-efficacy	339	2.83 ± 0.72	82	2.87 ± 0.68	257	2.81 ± 0.73	244	2.79 ± 0.69
PA enjoyment	352	23.81 ± 5.65	89	24.88 ± 4.93	263	23.44 ± 5.84	250	24.04 ± 4.72
Sedentary enjoyment	351	4.64 ± 0.77	89	4.59 ± 0.74	262	4.66 ± 0.78	245	4.78 ± 0.70
Personal barriers	343	2.31 ± 0.77	90	2.18 ± 0.68	253	2.36 ± 0.8	246	2.39 ± 0.77
Organisation barriers	348	2.01 ± 0.73	88	2.01 ± 0.67	260	2.01 ± 0.75	243	2.02 ± 0.73
Family support	353	3.82 ± 1.00	89	3.91 ± 0.98	264	3.79 ± 1.01	251	3.82 ± 0.91
Friends support	357	3.43 ± 0.98	90	3.58 ± 0.91	267	3.38 ± 1	249	3.53 ± 0.91

^1^PA guidelines: At least 60 min of LTMVPA each day (i.e. all of the past seven days).

### Intervention effects

Table 2 presents the findings from analyses of 17 quantitative outcome measures: three HRQoL measures, two PA measures, and 12 potential mediators. The table includes results of 2-group analyses (condition: intervention vs control) and 3-group analyses (intervention completers i.e. school + community components, intervention non-completers i.e. school component only, control) on the differences between groups at follow-up with regard to each quantitative measure, with adjustment for corresponding baseline scores. For variables where significant differences were present, the direction and magnitude of significant pairwise group differences are also shown. In every case, when the analysis was repeated for the subsample who had provided weight and height data, with and without adjustment for age and BMI (results not tabulated), the adjustment made no substantive difference to the results. For most measures, there was no evidence of clustering of responses by school. However, personal and organisational/environmental barriers did provide significant clustering effects which were accounted for in the LMM.

**Table 2:** **Differences between groups at follow**-**up**, **with adjustment for baseline score**

**Variable**	**Group**	***n***	***p*-*value***	**Group mean difference**^**#**^	**Group**^**^**^	***n***	***p*-*value***	**Group mean differences**^**#**^		
				G1 - G2				G1 - G2	G1 - G3	G2 - G3
**Health**										
PedsQL – physical functioning	1. Intervention	340	0.005*	+3.0	1. Completers	90	0.021*			+3.1
	2. Control	243			2. Non-completers	250				
					3. Control	243				
PedsQL – psycho-social functioning	1. Intervention	337	0.001*	+3.8	1. Completers	89	0.003*			+4.3
	2. Control	242			2. Non-completers	248				
					3. Control	242				
PedsQL total score	1. Intervention	338	0.001*	+3.5	1. Completers	89	0.002*			+3.9
	2. Control	243			2. Non-completers	249				
					3. Control	243				
**Physical activity**										
PDPAR-24: MET-mins of LTMVPA	1. Intervention	136	0.241		1. Completers	25	0.293			
	2. Control	107			2. Non-completers	111				
					3. Control	107				
PDPAR-24: Mins of LTMVPA	1. Intervention	136	0.555		1. Completers	25	0.419			
	2. Control	107			2. Non-completers	111				
					3. Control	107				
Days out of past seven with at least 60 min LTMVPA	1. Intervention	351	0.132		1. Completers	89	0.152			
	2. Control	255			2. Non-completers	262				
					3. Control	255				
**Potential mediators**										
Perceived sports competence	1. Intervention	338	0.865		1. Completers	85	0.922			
	2. Control	257			2. Non-completers	253				
					3. Control	257				
Self-management strategies	1. Intervention	333	0.568		1. Completers	82	0.010*	+0.25		
	2. Control	246			2. Non-completers	251				
					3. Control	246				
Perceived behavioral control	1. Intervention	354	0.704		1. Completers	87	0.034*	+0.27	+0.18	
	2. Control	255			2. Non-completers	267				
					3. Control	255				
Outcome expectation	1. Intervention	340	0.654		1. Completers	84	0.328			
	2. Control	251			2. Non-completers	256				
					3. Control	251				
Outcome expectancy-value	1. Intervention	323	0.213		1. Completers	80	0.008*	+17.4	+18.4	
	2. Control	192			2. Non-completers	243				
					3. Control	192				
Self-efficacy	1. Intervention	321	0.502		1. Completers	78	0.006*	+0.28	+0.25	
	2.Control	232			2. Non-completers	243				
					3. Control	232				
PA Enjoyment	1. Intervention	338	0.648		1. Completers	84	0.208			
	2. Control	245			2. Non-completers	254				
					3. Control	245				
Sedentary enjoyment	1. Intervention	332	0.195		1. Completers	83	0.104			
	2. Control	241			2. Non-completers	249				
					3. Control	241				
Personal barriers	1. Intervention	320	0.018*	−0.13	1. Completers	83	0.004*	+0.19		−0.18
	2. Control	239			2. Non-completers	237				
					3. Control	239				
Organisation barriers	1. Intervention	320	0.909		1. Completers	83	0.113			
	2. Control	241			2. Non-completers	247				
					3. Control	241				
Family support	1. Intervention	338	0.122		1. Completers	89	0.017*	+0.22	+.26	
	2. Control	240			2. Non-completers	249				
					3. Control	240				
Friends support	1. Intervention	340	0.345		1. Completers	90	0.048*	+0.22		
	2. Control	236			2. Non-completers	250				
					3. Control	238				

^#^Significant differences between group mean scores (G) *p < 0.05.

^^^Completers = school component + community component; Non-completers = school component only.

#### HRQoL

The 2-group analysis (intervention vs control) showed that, after adjustment for baseline levels of PedsQL, the intervention group had significantly higher scores on all three PedsQL scores – physical functioning (adjusted M ± SE = 83.9 ± 0.7, p = .005), psychosocial (79.9 ± 0.8, p = .001) and total score (81.3 ± 0.7, p = .001) – than the control group (80.9 ± 0.8; 76.1 ± 0.9 and 77.8 ± 0.8 respectively), suggesting that the program positively influenced quality of life. Differences in PedsQL were also present in the 3-group analysis (intervention completers, intervention non-completers and control), whereby the intervention non-completers had significantly higher scores (84.0 ± 0.8, p = .021; 80.4 ± 0.9, p = .003; and 81.7 ± 0.8, p = .002 respectively) than the control group (80.9 ± 0.8, 76.1 ± 0.9 and 77.8 ± 0.8 respectively). The lack of a significant difference between intervention completers and the control group may be attributed to a combination of two factors: the fact that the intervention completers group had higher PedsQL scores at baseline than the other groups (possible ceiling effect) and the smaller sample size of the intervention completers group. Paired t-tests of changes over time within each group revealed a further aspect of the differences between intervention and control groups. All PedsQL scores decreased significantly in the control group (physical functioning: M = −3.3, p = .001; psychosocial functioning: M = −3.8, p < .001; total score: M = −3.6, p = .001), while there were no significant changes over time in the intervention group (physical functioning: M = −0.0, p = .992; psychosocial functioning: M = −0.3, p = .726; total score: M = −0.2, p = .791).

#### Physical activity

There was no statistically significant difference in either the 2-group or 3-group analysis for mins of LTMVPA, MET-mins of LTMVPA, or in the proportion meeting physical activity guidelines.

#### Potential mediator variables

There were no statistically significant differences between intervention and control groups among the potential mediator variables. However, the 3-group analyses showed that there were significant differences between groups on several potential mediator variables after adjustment for the corresponding measure at baseline. Specifically, intervention completers had significantly higher scores than both intervention non-completers and controls for perceived behavioural control (adjusted M ± SE: completers 4.05 ± 0.07; non-completers 3.84 ± 0.04; controls 3.87 ± 0.04, p = .034), outcome expectancy-value (completers 157.5 ± 5.3; non-completers 140.1 ± 3.0; controls 139.1 ± 3.4, p = .008), self-efficacy (completers 3.03 ± 0.08; non-completers 2.75 ± 0.05; controls 2.78 ± 0.05, p = .006) and family support (completers 3.90 ± 0.08; non-completers 3.67 ± 0.05; controls 3.63 ± 0.05, p = .017), and significantly higher scores than intervention non-completers, but not controls, for self-management strategies (completers 3.63 ± 0.07; non-completers 3.38 ± 0.04; controls 3.47 ± 0.04, p = .10), personal barriers (completers 2.57 ± 0.07; non-completers 2.38 ± 0.04; controls 2.56 ± 0.04, p = .004) and support of friends (completers 3.46 ± 0.08; non-completers 3.23 ± 0.05; controls 3.36 ± 0.05, p = .048). There were no significant differences with regard to enjoyment of PA or sedentary pursuits, perceived sports competence, or organisational/environmental barriers.

Table 3 presents the findings from logistic regression analyses of three dichotomous indicators: perceived level of PA (moderate or high v low), having met PA guidelines (at least 60 mins of MVPA per day) on each of the past seven days (yes v no) and sport club membership (member v non-member). The table includes results of 2-group analyses (condition: intervention vs control) and 3-group analyses (intervention completers i.e. school + community components, intervention non-completers i.e. school component only, control) on the differences between groups at follow-up, with adjustment for baseline values. For each indicator, when the analysis was repeated for the subsample who had provided weight and height data, with and without adjustment for age and BMI (results not tabulated), the adjustment made no substantive difference to the estimated group differences.

**Table 3:** **Analysis of dichotomous variables for groups at follow**-**up**, **with adjustment for baseline category**

**Variable**	**Group**	***n***	***p*-*value***	**Odds ratio**^**#**^	**Group**^**^**^	***n***	***p*-*value***	**Odds ratio**^**#**^
Self-reported PA level (moderate/high v low)	1. Intervention	358	0.977		1. Completers	90	0.578	
	2. Control (reference)	258			2. Non-completers	268	0.838	
					3. Control (reference)	258		
Met PA guidelines in past seven days	1. Intervention	351	0.772		1. Completers	89	0.466	
	2. Control (reference)	255			2. Non-completers	262	0.455	
					3. Control (reference)	255		
Sport club/leisure centre (member v non-member)	1. Intervention	355	0.057		1. Completers	88	0.878	
	2. Control (reference)	255			2. Non-completers	267	0.021*	1.66
					3. Control (reference)	255		

^#^Significant odds ratios *p < 0.05.

^^^Completers = school component + community component; Non-completers = school component only.

With regard to self-reported PA level and meeting PA guidelines, there were no statistically significant differences between intervention and control groups, nor between completers, non-completers and controls. With regard to sports club/leisure centre membership, after adjustment for baseline levels of sports club membership, the difference between intervention and control groups in the odds of belonging to a sports club/leisure centre were not quite statistically significant (p = .056), but there were significant differences between non-completers and controls (OR = 1.66; p = .021), with the intervention non-completers group more likely to be members of sports clubs/leisure centres. Again, the lack of a significant difference between intervention completers and the control group can be attributed to a combination of two factors: the fact that the intervention completers group had higher percentage of sports club membership at baseline than the other groups (likely ceiling effect) and the smaller sample size of the intervention completers group. McNemar tests of changes over time within each group revealed that the percentage of sports club members decreased, though not significantly, in both the intervention completer group (baseline 60.2%; follow-up 56.8%; p = .648) and the control group (baseline 59.6%; follow-up 55.7%; p = .220), and increased significantly in the intervention non-completer group (baseline 53.2%; follow-up 60.3%; p = .016).

## Discussion

This study evaluated the effectiveness of a school-community linked PA-promotion intervention program on increasing HRQoL, PA, and a range of potential mediators of PA among adolescent girls living in low-SES regional and rural communities. Studies such as this which are realistic in their scope are important as they provide the real-world implementation evidence to inform public policy and professional practice. However, they are very challenging to implement as they require the engagement of multiple stakeholders with varying objectives and capacities over a considerable time period [60].

The program design was informed by learnings from similar randomised controlled trials [8,9]. The program included: formative research to inform the program design; the socioecological model as the overarching theoretical framework – underpinned by social cognitive theory, capacity building strategies and educational theory; and attempted to create direct links between school and community PA opportunities to sustain PA participation [19]. We know that studies consisting of both physical and cognitive components are more likely to have significant positive intervention results [12]. This study incorporated both of these elements and there were a number of significant positive results.

In terms of HRQoL, previous research has shown that HRQoL decreases during adolescence, especially among girls [61]. In this study, such a decrease was observed in the control group, while the intervention group maintained its baseline levels of HRQoL. The intervention may have had a protective effect on the intervention group’s HRQoL, which is a positive outcome of the study. Specifically, the group difference between intervention and control for the three PedsQL scores ranged from +3.0 to +3.8, which is approaching minimal clinically important differences in PedsQL scores for youth without and with type 2 diabetes (+5.41 for total score) [62].

There were no significant changes in (self-reported) PA levels. Other multi-component interventions with school- and community strategies have resulted in only modest improvements in girls’ PA and have suggested that the length of time girls are exposed to the intervention may affect PA [8]. A recent systematic review of PA interventions in the school setting [12] reported that medium-term studies (i.e. four to 12 months) reported significant differences in PA levels in favour of the intervention group more frequently (68.6%) than short-term (i.e. less than three months, 47.4%) or long-term studies (i.e. 13 or more months, 45.0%). However, long-term school-based interventions have also reported negative effects on student attitude towards PA [12]. In addition, interventions were likely to achieve positive PA outcomes if the intervention was delivered four or more times per week [12] and whole of school approaches have also been shown to be effective [9].

The length of the intervention in this study may have been insufficient to achieve changes in PA. The intervention in this study was modest and realistic in scope, and specifically focused on developing formal and evidence-based strategies to link schools and community organisations for sustained participation. The school component was implemented as two 6-session units delivered during the scheduled PE class time, generally one session per week for six weeks for each unit. Given this focus on the PE setting, the intervention was subject to timetable and curriculum constraints. Others have reported institutional barriers to the provision of PE in Australian schools including restricted timetabling of PE and lack of access to facilities, equipment and suitable teaching spaces [63]. In addition, PE is often organised with a predisposition towards team games and development of sport skills [64], and therefore, a limited range of physical activities, which are not necessarily lifelong activities [65,66]. The intervention in this study included a traditional team sport (football), a lifestyle sport (tennis) and a range of lifestyle physical activities (leisure centre activities) that were linked to local facilities and programs to add value to the existing school PE program, which has been recommended by others [67]. Further, at the conclusion of the intervention, only a quarter of students in the intervention group reported attending one of the community-based sports programs outside of school. Developing meaningful and sustained linkages between schools and community settings for PA across a range of PE curriculum and activity areas may result in the achievement of better outcomes.

In terms of becoming a sports club or leisure centre member, after adjustment for sports club/leisure centre membership at baseline, non-completers were more likely to belong to a sports club at follow-up, not only compared to controls, but also compared to completers. The latter counter-intuitive result may be attributable to a number of factors. There was a higher proportion of sports club/leisure centre membership at baseline among those who went on to complete the program than among non-completers. It may also be that the intervention raised awareness among the non-completer group about joining a sports club/leisure centre that was not linked to the intervention per se. The intervention was limited to two sports and one recreational organisation and as a consequence factors such as personal preference, access and peer influence may have affected this outcome. Again, the notion of developing a range of linkages between schools and community settings for PA in various PE curriculum and activity areas may result in the provision of student choice and potentially better outcomes.

The intervention also appeared to have positive effects on a range of intra-personal capacities (i.e. self-efficacy, self-management, perceived behavioural control, outcome expectancy-value) and inter-personal factors (i.e. support from family and friends), as improvements favoured intervention completers over non-completers, and to a lesser degree over controls. Few PA interventions have assessed mediators of PA behaviour among youth [68] and studies that examine the mediators of behaviour change in interventions are required [69]. Several studies have reported that mediators of PA, such as self-efficacy, partially mediate the effects of an intervention on PA [68,69]. In our study, while PA did not change, there were positive differences between intervention and control groups with regard to both HRQoL and previously identified PA mediators. These anomalous results may in part be attributable to lower power from the smaller sample sizes for the PDPAR variables, but may also reflect the existence of pathways to HRQoL in this intervention other than via the quantity of PA alone.

There were no significant changes with regard to enjoyment of PA or non-PA pursuits, perceived sport competence or perceived organisational/environmental factors. In terms of perceived sports competence, the sports of tennis and football adopted ‘Game Sense’ as the pedagogical approach to the intervention curriculum. Specifically, the intervention drew on Game Sense pedagogies to avoid traditional ‘command-orientated’ and ‘teacher/coach-directed’ pedagogies that tend to be more characteristic of masculine approaches to teaching [70] and were identified as a barrier in the ethnographic fieldwork [19]. Further, the ethnographic fieldwork highlighted that girls tended to have strongly entrenched ideas about their own physical ability, which shaped their attitudes towards, and participation in, PE and out-of-school sport [20]. Game Sense places participation in games as the foundation from which teaching and skill refinement proceeds [71] and has been shown to strongly impact on students’ learning, especially among girls and low skill-level students [72]. Process evaluation of the implementation of the program revealed that the understanding of, and commitment to, the intention of a Game Sense approach varied among both teachers and coaches [37,73]. In particular, there was a widely held perception that fundamental motor skills were a prerequisite to game play, which indicates that in many cases the intervention may not have been implemented as intended. Whilst strategies such as professional development workshop and cooperative delivery model were employed to empower teachers and coaches further work is required in the provision of sport and physical activities for adolescent girls [73]. The research team acknowledge critiques of a multi-activity sports approach to physical education [74,75] and particularly for the decontextualized approach to skill learning, implementation of the intervention program relied on the cooperation of teachers and coaches. It was decided that a ‘repackaging’ of a unit of work in PE might help to identify the merits of such an approach that could potentially inform teaching and learning practices in the physical education program more broadly.

The lack of significant differences in PedsQL scores between completers and the control group may be a consequence of the fact that completers had higher PedsQL scores at baseline than non-completers and control groups, which may mean significant changes were harder to achieve. Specifically, the health and PA profiles of completers at baseline were comparable to population norms for PedsQL scores [76] and proportion of students meeting PA guidelines of MVPA for 60 minutes or more in the past 7 days [77]. In comparison, the control groups had lower scores compared to a healthy sample of children aged 5 – 18 years for all three PedsQL measures reported by Varni and colleagues (total score: 83.00 ± 14.79; Physical functioning 84.41 ± 17.26; and psychosocial functioning: 82.38 ± 15.51), whilst non-completers had lower PedsQL scores than the healthy sample for psychosocial functioning and total score, but not physical functioning [76].

Similarly, in terms of the proportion of students meeting PA guidelines at baseline, the intervention group (11.2%) were comparable to an Australian sample of students from 2005, in which 12.7% of students aged 12 – 13 and 12.1% of students aged aged14 – 15 were engaged in at least 60 minutes of MVPA each day in the previous week [77]. In comparison, the control group had a very low proportion (5.8%) of students meeting PA guidelines [77].

The socio-ecological model was the overarching theoretical framework – underpinned by individual through to organisational strategies designed to develop linkages between school and community PA opportunities in order to improve PA participation [19]. Some aspects of the program were not fully implemented as intended, such as the student-centred pedagogical approach (Game Sense) and self-management strategies, which is likely to have had a negative impact on the intended dose of the intervention [37]. Program implementation barriers within the school setting were related to a perception that fundamental motor skills were a prerequisite to game play, a lack of experience among program deliverers with the teaching approach, and complex organisational barriers like school timetabling, and are discussed in detail elsewhere [37,73].

### Strengths and limitations

The strengths of this study include the prospective and controlled study design, which allowed the effects of the intervention to be assessed comparatively and longitudinally over time. With regard to limitations, studies of this population cohort are very hard to conduct, particularly in light of the ethics requirement of Australian education authorities to obtain specific ‘opt-in’ parental consent, which is exacerbated by the necessity to communicate with parents only indirectly in writing via the school and the students themselves. The research team were dependent on the efforts of teachers with many competing priorities to facilitate and promote recruitment and retention, amid the complexities of school operations and scheduling. Consequently, limitations to this study include moderate consent/recruitment and retention rates in the intervention group, and low consent/recruitment, albeit offset by a higher retention rate, in the control group. A potential consequence of the low to moderate recruitment rate is self-selection bias; if more physically active girls were more motivated to participate in the study, the study sample would not be representative of the whole population of adolescent girls. A potential effect on longitudinal analyses of moderate retention rates is the possibility of further bias due to less physically active participants being more likely to drop out of the study. A further consequence is reduced statistical power, due to failure to achieve the design target sample sizes. Despite (and perhaps because of) these unavoidable limitations, there have been few interventions and little evaluative research in this domain, particularly longitudinal research, and so our findings, interpreted with appropriate caution, are important.

Objective measures of PA would have strengthened the assessment of potential outcomes, as self-report measures provide low to moderate validity and participants may have difficulties recalling information [47]. The self-report approach was used because diaries are considered appropriate to capture a large participant base at low cost [38] and are relatively unobtrusive and quick to administer compared to objective measures. In addition, it was over-ambitious to attempt to fit the lengthy survey form, especially the PDPAR-24 component, into school timetable slots, which resulted in many incomplete diaries and hence loss of statistical power with regard to the measurement of LTMVPA and MET-weighted LTMVPA.

## Conclusion

In conclusion, the school community-linked intervention resulted in a number of positive outcomes. These positive outcomes arose from what was effectively a modest intervention that was focused on formal strategies to link schools with community organisations for sustained participation. The school component of the program appeared to contribute to maintaining HRQoL; whilst those students who completed the community component developed a range of intra-personal capacities such as self-efficacy and inter-personal factors including support from family, which support participation in PA. It also appeared that the intervention increased awareness of sports clubs/leisure centres in the community, as the rate of membership of these community organisations significantly increased among non-completers. Developing meaningful and sustained linkages between schools and community settings for PA across a range of PE curriculum and activity areas may result in the achievement of better outcomes.

In summary, we observed a protective effect on HRQoL as a result of participation in this modest intervention program, which was independent of any change in PA. This was unexpected and may be attributed to an enhanced sense of control and self-efficacy developed in response to the intervention program that was designed to meet the needs of the participants.

## Competing interests

The authors declare that they have no competing interests.

## Authors’ contributions

WP and AT were co-principal investigators on this study. They led the design of the study and obtained funding. WP was the chief investigator and oversaw all aspects; whilst AT led the development of the school component. John Smyth was also a co-principal investigator on the study and led the ethnographic fieldwork, which has been reported elsewhere. MC was the project manager and was responsible for data collection, implementation, and analysis. AT and AM designed the intervention materials and provided professional development to schools. JH oversaw statistical design, analysis and reporting; whilst RE and AM provided expertise in sport and recreation and physical education respectively. All authors contributed to the design of the study, critically commented on the manuscript, and read and approved the final manuscript.

## Pre-publication history

The pre-publication history for this paper can be accessed here:

http://www.biomedcentral.com/1471-2458/14/649/prepub
